# Involvement of the leptin-adiponectin axis in inflammation and oxidative stress in the metabolic syndrome

**DOI:** 10.1038/s41598-017-06997-0

**Published:** 2017-07-26

**Authors:** Gema Frühbeck, Victoria Catalán, Amaia Rodríguez, Beatriz Ramírez, Sara Becerril, Javier Salvador, Piero Portincasa, Inmaculada Colina, Javier Gómez-Ambrosi

**Affiliations:** 1 Metabolic Research Laboratory, Clínica Universidad de Navarra, Pamplona, Spain; 2 CIBER Fisiopatología de la Obesidad y Nutrición (CIBEROBN), Instituto de Salud Carlos III, Pamplona, Spain; 3 Obesity and Adipobiology Group, Instituto de Investigación Sanitaria de Navarra (IdiSNA), Pamplona, Spain; 4 Department of Endocrinology & Nutrition, Clínica Universidad de Navarra, Pamplona, Spain; 5 Clinica Medica “A. Murri”, Department of Biomedical Sciences and Human Oncology, University of Bari Medical School, Policlinico Hospital, Bari, Italy; 6 Department of Internal Medicine, Clínica Universidad de Navarra, Pamplona, Spain

## Abstract

The aim of the present work was to study whether the leptin-adiponectin axis may have a pathophysiological role in the increased systemic inflammation and oxidative stress observed in patients with the metabolic syndrome (MS). Leptin, adiponectin, and markers of inflammation and oxidative stress were measured in a sample of 140 Caucasian subjects (74 males/66 females), aged 28–82 years, 60 with and 80 without the MS. Total concentrations of adiponectin as well as its multimeric forms HMW, MMW and LMW were significantly lower in individuals with the MS. The ratio adiponectin/leptin, a marker of dysfunctional adipose tissue, was dramatically decreased in the MS group. Systemic oxidative stress, as evidenced by levels of thiobarbituric acid reactive substances (TBARS), as well as markers of inflammation such as serum amyloid A (SAA), C-reactive protein (CRP) and osteopontin were significantly increased in subjects with the MS. Total adiponectin concentrations were negatively correlated with levels of TBARS and CRP levels. Furthermore, the ratio adiponectin/leptin was negatively correlated with SAA concentrations as well as with CRP levels. We concluded that a dysfunctional adipose tissue as suggested by a low adiponectin/leptin ratio may contribute to the increased oxidative stress and inflammation, hallmarks of the MS.

## Introduction

Excess adiposity favors the development of cardiometabolic alterations such as type 2 diabetes, hypertension, and dyslipidemia, leading to an increase in morbidity^1^. These metabolic alterations frequently cluster together in a disorder known as metabolic syndrome (MS)^2^ contributing to cardiovascular morbidity and mortality^3^. The International Diabetes Federation estimates that 25% of the adult population worldwide suffers from the MS, which is common and is increasing in the industrialized societies^4^. Given the overall burden of the MS and its cardiometabolic consequences, further research is necessary to unravel the complex pathways involved in its etiopathogenesis.

Adipose tissue secretes a wide variety of biologically active molecules thus representing an extremely active endocrine organ^5^. These secreted proteins, collectively called adipokines are known to be involved in the pathophysiological link between increased adiposity and cardiometabolic alterations^5^. It has been reported that the MS may induce or even may be caused by increased systemic inflammation and oxidative stress in relation with an altered adipokine secretion^6^. Leptin is an adipokine primarily produced by adipose tissue in proportion to the amount of body fat involved in the regulation of energy homeostasis, neuroendocrine function, hematopoiesis, angiogenesis, and reproduction, among others^7^. Adiponectin is another adipokine expressed almost exclusively in adipose tissue^8^. Plasma adiponectin concentrations are decreased in obese patients as well as in patients with the MS^9^. Adiponectin increases insulin sensitivity and exerts also anti-inflammatory actions^10^. This adipokine exists in plasma in 3 major oligomeric forms: a low-molecular-weight (LMW) trimer, a middle-molecular-weight (MMW) hexamer, and a high-molecular-weight (HMW) 12- to 18-mer^11^. Previous studies show that HMW adiponectin better predicts insulin resistance and the MS in humans^11, 12^. Moreover, the adiponectin/leptin ratio has been suggested as a maker of adipose tissue dysfunction and correlates with insulin resistance more closely than adiponectin or leptin alone or even HOMA, a surrogate of insulin resistance^13^. Reportedly, this ratio decreases with increasing number of metabolic risk factors reflecting the functionality of adipose tissue, having been proposed as a predictive marker for the MS^14^.

A wide number of studies have reported that a state of low-grade chronic inflammation and oxidative stress may be involved in the development of the MS^15–19^. Concentration of thiobarbituric acid reactive substances (TBARS) in blood is classically used as a marker of systemic oxidative stress and has been related to the MS^20^. On the other hand, classical markers of low-grade chronic inflammation, such as C-reactive protein (CRP) have been associated with the presence of the MS and its associated cardiometabolic risk^21^. However, in recent years other interesting proinflammatory factors, such as serum amyloid A (SAA) or osteopontin (OPN), have been related to the development of cardiometabolic alterations^22, 23^. However, its role in the etiology of the MS has not been fully clarified.

The aim of the present work was to analyze whether the leptin-adiponectin axis may have a pathophysiological role in the increase in oxidative stress and inflammation observed in patients with the MS.

## Results

Clinical characteristics of the patients enrolled in the study are summarized in Table 1. No statistically significant differences for gender distribution or age were found between the groups. Body weight, body mass index (BMI), body adiposity and waist circumference were significantly higher (*P* < 0.001) in subjects with the MS. Patients with the MS were more insulin resistant than control individuals as evidenced by the increased concentrations of glucose (*P* < 0.001) and insulin (*P* < 0.001), and the higher homeostatic model assessment (HOMA) and lower quantitative insulin sensitivity check index (QUICKI) (*P* < 0.001 for both). Patients with the MS exhibited higher circulating concentrations of triglycerides (*P* < 0.001), while high-density lipoprotein-cholesterol (HDL-C) was reduced (*P* < 0.001). Uric acid, white blood cell (WBC) as well as hepatic enzymes were increased (*P* < 0.01) in subjects with the MS (Table 1).

**Table 1:** Demographic and biochemical characteristics of the individuals at enrollment.

	Without MS	With MS	*P*
n	80	60	
Sex, M/F	40/40	34/26	0.495
Age, y	58.8 ± 12.2	61.1 ± 9.0	0.221
Weight, kg	75 ± 16	93 ± 13	<0.001
BMI, kg/m^2^	27.4 ± 5.5	33.8 ± 4.4	<0.001
Body adiposity, %	34.6 ± 7.5	41.0 ± 7.3	<0.001
Waist circumference, cm	92 ± 14	109 ± 9	<0.001
SBP, mm Hg	136 ± 24	143 ± 18	0.053
DBP, mm Hg	80 ± 12	84 ± 10	0.029
Glucose, mg/dL	94 ± 13	114 ± 19	<0.001
Insulin, μU/mL	4.2 ± 3.3	13.6 ± 10.2	<0.001
HOMA	1.0 ± 0.8	3.9 ± 3.3	<0.001
QUICKI	0.408 ± 0.053	0.327 ± 0.035	<0.001
Triglycerides, mg/dL	88 ± 33	154 ± 90	<0.001
Cholesterol, mg/dL	215 ± 35	209 ± 48	0.331
LDL-C, mg/dL	135 ± 35	126 ± 37	0.148
HDL-C, mg/dL	63 ± 17	50 ± 14	<0.001
Uric acid, mg/dL	5.2 ± 1.5	6.2 ± 1.3	<0.001
WBC, 10^6^ cells/mL	6.2 ± 1.8	7.1 ± 1.6	0.007
ALT, IU/L	14 ± 4	18 ± 9	<0.001
AST, IU/L	14 ± 17	23 ± 15	0.009
AST/ALT ratio	1.06 ± 0.52	0.76 ± 0.29	<0.001
γ-GT, IU/L	18 ± 11	41 ± 38	<0.001
Creatinine, mg/dL	0.92 ± 0.17	0.90 ± 0.26	0.553

Data presented as mean ± SD. Individuals were matched by age and sex. MS, metabolic syndrome; BMI, body mass index; SBP, systolic blood pressure; DBP, diastolic blood pressure; HOMA, homeostatic model assessment; QUICKI, quantitative insulin sensitivity check index; LDL-C, low-density lipoprotein-cholesterol; HDL-C, high-density lipoprotein-cholesterol; WBC, white blood cells; ALT, alanine aminotransferase; AST, aspartate aminotransferase; γ-GT, γ-glutamyltransferase. Differences between groups were analyzed by two-tailed unpaired Student’s *t* tests. Differences in gender distribution were analyzed by χ^2^ analysis.

**Figure 1:**
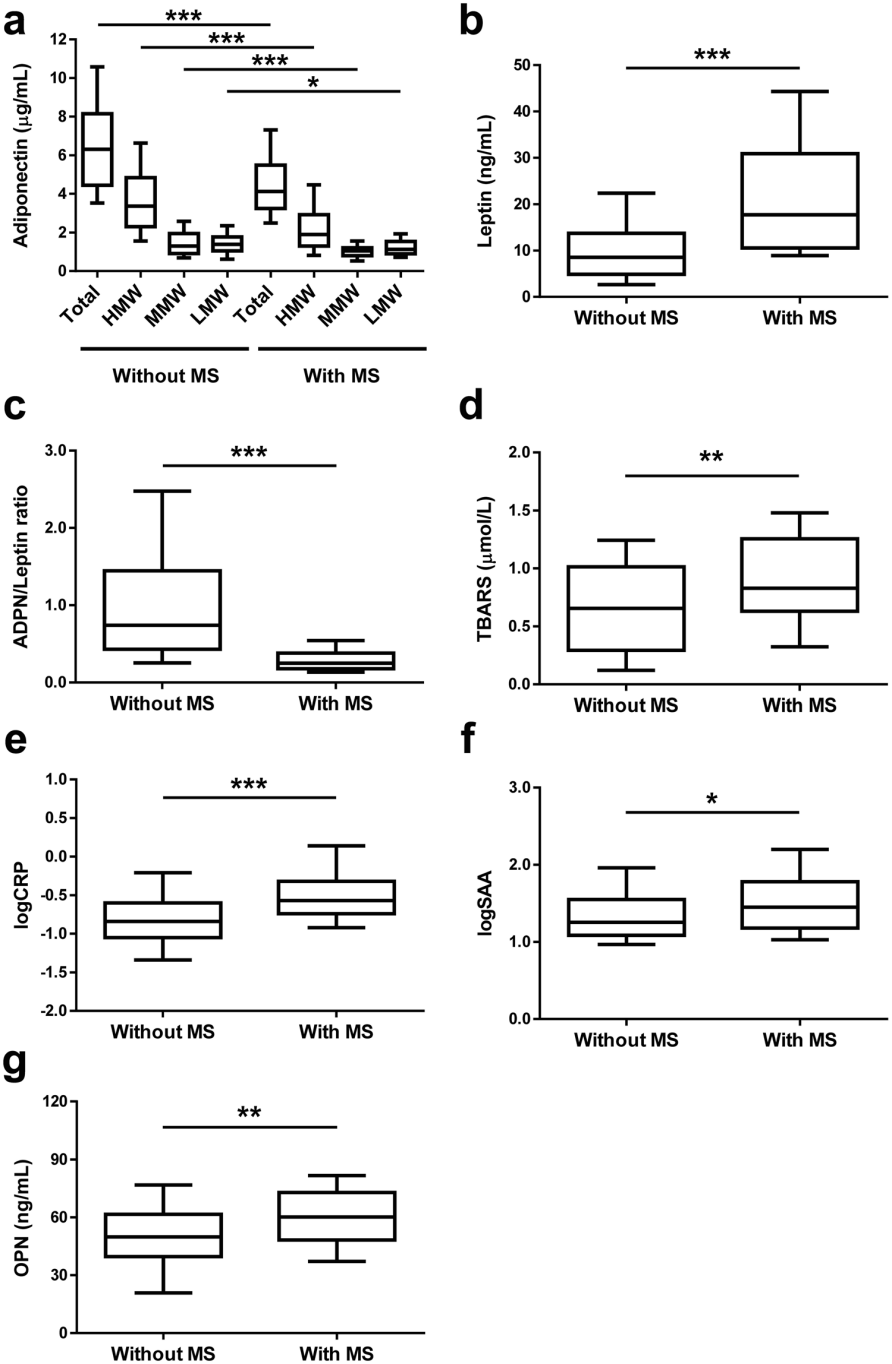
Circulating adiponectin (ADPN) concentrations are decreased in patients with the metabolic syndrome (MS) in relation with increased oxidative stress and inflammation. Total and multimeric adiponectin (**a**), leptin (**b**), ADPN/leptin ratio (**c**), thiobarbituric acid reactive substances (TBARS, **d**), C-reactive protein (CRP, e), serum amyloid A (SAA, f) and osteopontin (OPN, **g**) concentrations in subjects with or without the MS. *Box represents* interquartile range and median inside, with whiskers showing 10/90 percentile. Differences between groups were analyzed by two-tailed unpaired Student’s t tests. *P < 0.05, **P < 0.01 and ***P < 0.001 for with MS vs without MS. CRP and SAA were logarithmically transformed due to non-normal distribution.

**Figure 2:**
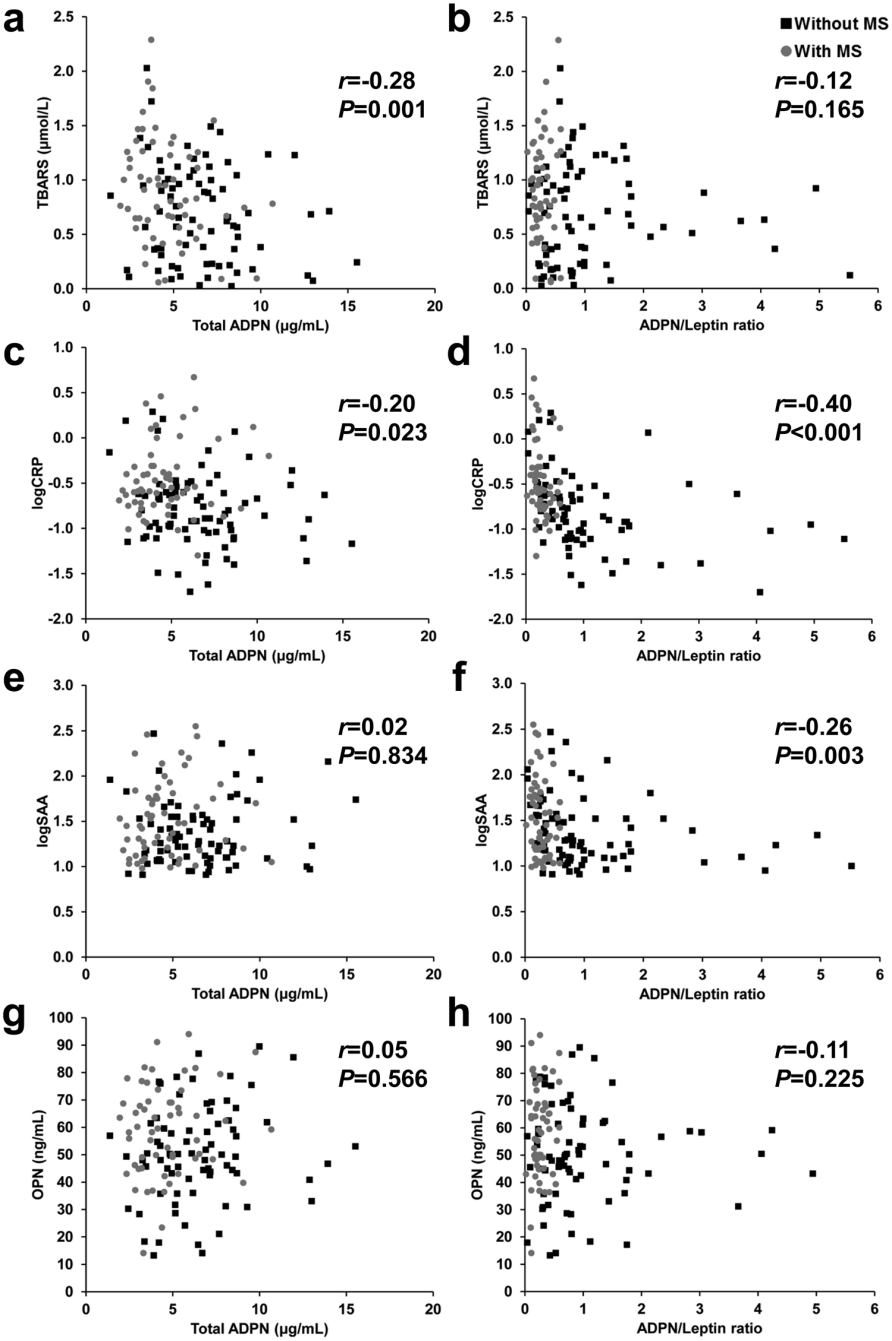
Scatter diagrams showing the correlations between the circulating concentrations of total adiponectin and the ADPN/leptin ratio with the levels of TBARS (**a** and **b**), CRP (**c** and **d**), SAA (**e** and **f**) and osteopontin (OPN) (**g** and **h**). Pearson’s correlation coefficient and *P* values are indicated. CRP and SAA were logarithmically transformed due to non-normal distribution.

Total as well as HMW, MMW (*P* < 0.001 for all) and LMW (*P* < 0.05) adiponectin concentrations were significantly lower in individuals with the MS (Fig. 1a). In contrast, leptin levels were significantly higher (*P* < 0.001) in subjects with the MS (Fig. 1b). The ratio adiponectin/leptin, a marker of dysfunctional adipose tissue^14^, was dramatically decreased (*P* < 0.001) in the MS group (Fig. 1c). Systemic oxidative stress, as evidenced by levels of TBARS, was significantly increased (*P* < 0.01) in patients with the MS (Fig. 1d). Markers of inflammation such as SAA (*P* < 0.05, Fig. 1e), CRP (*P* < 0.001, Fig. 1f) and OPN (*P* < 0.01, Fig. 1g) were more elevated in subjects with the MS as compared with individuals without the MS. Total adiponectin concentrations were negatively correlated with levels of TBARS (*r* = −0.28, *P* = 0.001; Fig. 2a) and CRP concentrations (*r* = −0.20, *P* = 0.023; Fig. 2c). Furthermore, the adiponectin/leptin ratio was negatively correlated with SAA concentrations (*r* = −0.26, *P* = 0.003; Fig. 2f) as well as with CRP concentrations (*r* = −0.40, *P* < 0.001; Fig. 2d). Circulating concentrations of OPN were not correlated with adiponectin levels (Fig. 2g) nor with the adiponectin/leptin ratio (Fig. 2h).

In order to evaluate the degree of association of the markers of inflammation and oxidative stress studied, with the anthropometric and biochemical variables, a bivariate correlation analysis was carried out (Table 2). Levels of TBARS were correlated with gender, age, weight, uric acid as well as markers of glucose and lipid metabolism. Concentrations of OPN were strongly correlated with age and markers of insulin resistance and, to a lesser extent, with estimators of total and central adiposity. Levels of SAA were correlated with gender, age and anthropometric indices, but not with metabolic variables. Finally, CRP and the adiponectin/leptin ratio showed strong correlations with anthropometric variables, glucose and lipid metabolism estimators as well as with markers of hepatic function.

**Table 2:** Univariate analysis of the correlation of markers of inflammation and oxidative stress with anthropometric and biochemical variables in the subjects included in the study.

	TBARS		logCRP		OPN		logSAA		ADPN/Leptin	
	*r*	*P*	*r*	*P*	*r*	*P*	*r*	*P*	*r*	*P*
Sex	−0.17	0.044	0.14	0.094	0.02	0.817	**0.29**	**<0.001**	−0.09	0.302
Age	**−0.32**	**<0.001**	0.17	0.044	**0.29**	**<0.001**	**0.33**	**<0.001**	0.03	0.735
Weight	0.19	0.023	**0.35**	**<0.001**	0.11	0.222	0.03	0.690	**−0.52**	**<0.001**
BMI	0.05	0.543	**0.50**	**<0.001**	0.10	0.246	0.24	0.005	**−0.60**	**<0.001**
Body adiposity	−0.10	0.262	**0.50**	**<0.001**	0.20	0.023	**0.43**	**<0.001**	**−0.57**	**<0.001**
WC	0.12	0.176	**0.46**	**<0.001**	0.20	0.024	0.17	0.042	**−0.59**	**<0.001**
SBP	−0.12	0.156	0.09	0.311	0.20	0.021	0.02	0.796	−0.12	0.180
DBP	−0.02	0.857	0.20	0.017	0.15	0.077	0.07	0.406	**−0.33**	**<0.001**
Glucose	0.11	0.197	0.19	0.028	0.11	0.195	0.12	0.158	−0.18	0.045
Insulin	0.17	0.047	0.25	0.004	**0.32**	**<0.001**	0.14	0.114	**−0.33**	**<0.001**
HOMA	0.19	0.033	0.25	0.004	**0.30**	**<0.001**	0.15	0.091	**−0.31**	**<0.001**
QUICKI	−0.22	0.010	**−0.34**	**<0.001**	−0.27	0.002	−0.16	0.073	**0.48**	**<0.001**
Triglycerides	**0.41**	**<0.001**	0.18	0.031	0.07	0.419	0.03	0.750	**−0.30**	**<0.001**
Cholesterol	0.25	0.004	−0.04	0.676	−0.03	0.725	−0.02	0.787	−0.01	0.879
LDL-C	0.28	0.001	0.02	0.799	−0.01	0.960	−0.06	0.519	−0.01	0.873
HDL-C	**−0.32**	**<0.001**	−0.27	0.001	−0.07	0.436	0.04	0.669	**0.31**	**<0.001**
Uric acid	0.28	0.001	0.20	0.019	0.10	0.247	0.04	0.686	−0.25	0.005
WBC	0.07	0.420	**0.38**	**<0.001**	0.04	0.647	0.15	0.089	−0.27	0.003
ALT	0.09	0.296	0.27	0.002	0.09	0.289	0.05	0.564	−0.25	0.006
AST	−0.12	0.189	**0.34**	**<0.001**	0.17	0.061	0.13	0.152	−0.14	0.128
AST/ALT ratio	−0.19	0.032	−0.11	0.238	−0.05	0.590	0.03	0.716	0.18	0.047
γ-GT	0.11	0.213	**0.30**	**<0.001**	0.13	0.143	0.08	0.380	−0.18	0.059
Creatinine	0.06	0.517	0.06	0.47	0.07	0.448	0.11	0.202	0.04	0.674

TBARS, thiobarbituric acid reactive substances; CRP, C-reactive protein; OPN, osteopontin; SAA, serum amyloid A; ADPN, adiponectin; BMI, body mass index; WC, waist circumference; SBP, systolic blood pressure; DBP, diastolic blood pressure; HOMA, homeostatic model assessment; QUICKI, quantitative insulin sensitivity check index; LDL-C, low-density lipoprotein-cholesterol; HDL-C, high-density lipoprotein-cholesterol; WBC, white blood cells; ALT, alanine aminotransferase; AST, aspartate aminotransferase; γ-GT, γ-glutamyltransferase. Data are Pearson’s correlation coefficients and associated *P* values. For correlation with gender, male = 0 and female = 1 was used. Those correlations with a *P* lower than 0.001 are highlighted in bold.

## Discussion

The major findings of the present study are that total adiponectin and its multimeric forms were decreased in patients with the MS showing a significant negative correlation with markers of systemic inflammation and oxidative stress, and that the ratio adiponectin/leptin was negatively correlated with systemic inflammation. In addition, we show for the first time that SAA and OPN, novel obesity-associated markers of inflammation, are both increased in individuals with the MS.

We aimed to study whether the leptin-adiponectin axis is involved in the inflammation and oxidative stress associated with the presence of the MS. Patients with the MS in the present work exhibited increased levels of TBARS, evidencing higher levels of systemic oxidative stress, as compared to subjects without the MS, as well as increased concentrations of markers of inflammation such as CRP and SAA in agreement with previous studies^24, 25^. It has been reported that the MS is associated with increased levels of CRP, and the association and influence of this marker appeared to be cumulative; i.e. the higher the number of MS components, the higher levels of CRP^26^. Moreover, SAA has been shown to be closely associated with obesity^27, 28^ playing a critical role in local and systemic inflammation directly linking obesity with the development of insulin resistance and atherosclerosis^22^, therefore representing a potential molecular mediator in the development of the MS.

The presence of the MS was also accompanied by increased levels of OPN. Previous studies have reported elevated levels of OPN in obesity^29, 30^, but to our knowledge this is the first study linking increased levels of OPN with the MS. We have previously shown that OPN expression is dramatically increased in visceral adipose tissue in obesity and heavily involved in the obesity-associated metabolic derangements^29, 31^. Obesity-associated OPN overexpression in adipose tissue seems to be mediating the recruitment of macrophages and the development of inflammation and fibrosis in visceral adipose tissue^31, 32^. Although the molecular mechanisms involved have not been fully elucidated, our results suggest that OPN may be playing a pathophysiological role in the etiology of the MS^23^.

Decreased adiponectin levels or adiponectin signaling may serve as an upstream pathway of increased oxidative stress and inflammation in the development of the MS^33^. In our study, subjects with the MS showed reduced circulating concentrations of total adiponectin as well as its multimeric forms. In this sense, previous studies have shown that HMW adiponectin may serve as the most active multimeric form of adiponectin being a better indicator of insulin resistance than total adiponectin^12^. We found a negative correlation of adiponectin with markers of oxidative stress and inflammation confirming the protective effect of adiponectin against these MS-associated cardiometabolic derangements^11^. However, the correlations of HMW with cardiometabolic markers were very similar to those of total adiponectin. The adiponectin/leptin ratio has been reported to correlate with insulin resistance more closely than adiponectin or leptin alone or even HOMA^13^. This ratio decreases with increasing number of metabolic risk factors reflecting the functionality of adipose tissue, having been proposed as a predictive marker for MS^14^. In our study, a low adiponectin/leptin ratio was associated with high levels of markers of inflammation such as SAA and CRP. A lower adiponectin/leptin ratio may indicate that the high levels of leptin due to the leptin resistance characteristics of obesity and the MS^34^ is not been able to appropriately upregulate adiponectin expression and/or secretion, as evidenced in previous animal studies from our group^35^ and others^36^. Therefore, a dysfunctional adipose tissue as suggested by a low adiponectin/leptin ratio represents a hallmark of the MS with increased proinflammatory factors as potential pathophysiological mediators in its development, confirming the important role played by the leptin-adiponectin axis in this cardiometabolic condition. The lack of association of OPN, a molecule deeply involved in the development of obesity-associated insulin resistance as evidenced by previous works^23, 29, 31^ and the correlations observed in the present study, with the adiponectin/leptin ratio suggest the potential involvement of other factors secreted by the adipose tissue in inflammation and oxidative stress^37, 38^. For example, TNF-α, IL-1, IL-6, IL-8 and MCP-1 have been proposed as mediators of the expanded adipose tissue-mediated increase in systemic inflammation and oxidative stress^39–41^.

In summary, the MS is accompanied by a chronic pro-inflammatory state and increased oxidative stress. A negative correlation of adiponectin levels with markers of inflammation and oxidative stress was found. A dysfunctional adipose tissue as suggested by a low adiponectin/leptin ratio may contribute to the increased oxidative stress and inflammation, hallmarks of the MS. Moreover, SAA and OPN emerge as mediators in the development of MS therefore representing novel therapeutic targets for the treatment of this cardiometabolic disturbance.

## Materials and Methods

### Subjects

A sample of 140 Caucasian subjects (74 males/66 females), aged 28–82 years (mean ± SD, 60 ± 11y) matched for age, with similar socio-economical characteristics, recruited from patients visiting the Department of Endocrinology & Nutrition and the Department of Internal Medicine of the Clínica Universidad de Navarra with complete anthropometric measurements and analytics was enrolled. The presence or absence of the MS was based on the harmonized criteria^2^. Individuals with signs of infection were excluded. The experimental design was approved by the Research Ethics Committee of the University of Navarra and the study was performed in accordance with the ethical standards as laid down in the Declaration of Helsinki and its later amendments. Volunteers gave their informed consent to participate in the study.

### Anthropometric measurements

The anthropometric determinations as well as the blood sample extraction were performed on a single day. Height was measured to the nearest 0.1 cm with a Holtain stadiometer (Holtain Ltd., Crymych, UK), while body weight was measured with a calibrated electronic scale to the nearest 0.1 kg with subjects wearing a swimming suit and cap. Waist circumference was measured at the midpoint between the iliac crest and the rib cage on the midaxillary line. Blood pressure was measured after a 5-minute rest in the semi-sitting position with a sphygmomanometer. Blood pressure was determined at least 3 times at the right upper arm and the mean was used in the analyses. Percentage of body fat was estimated using the Clínica Universidad de Navarra-Body Adiposity Estimator (CUN-BAE)^42^.

### Serum biochemistry

Blood samples were collected after an overnight fast in the morning in order to avoid potential confounding influences due to hormonal rhythmicity. Plasma glucose was analyzed by an automated analyzer (Roche/Hitachi Modular P800, Basel, Switzerland) as previously described^1^. Insulin was measured by means of enzyme-amplified chemiluminescence assay (Immulite 2000, Siemens AG, Erlangen, Germany). Indirect measures of insulin resistance and insulin sensitivity were calculated by using HOMA and QUICKI, respectively. Total cholesterol and TG concentrations were determined by enzymatic spectrophotometric methods (Roche). Serum HDL-C was quantified by a colorimetric method in a Beckman Synchron^®^ CX analyzer (Beckman Instruments, Ltd., Bucks, UK). Low-density lipoprotein-cholesterol (LDL-C) was calculated by the Friedewald formula. WBC count was measured using an automated cell counter (Beckman Coulter, Fullerton, CA, USA). Uric acid, alanine aminotransferase (ALT), aspartate aminotransferase (AST), γ-glutamyltransferase (γ-GT), and creatinine were measured by enzymatic tests (Roche) in an automated analyzer (Roche/Hitachi Modular P800). The AST/ALT ratio was calculated as an indirect indicator of hepatic steatosis and fatty liver disease^43^. High-sensitivity CRP was measured using the Tina-quant CRP (Latex) ultrasensitive assay (Roche).Leptin was quantified by a double-antibody RIA method (Linco Research, Inc., St. Charles, MO, USA) as previously described^44^; intra- and inter-assay coefficients of variation were 5.0% and 4.5%, respectively. Adiponectin (ALPCO Diagnostics, Salem, NH, USA) and SAA^28^ (BioSource, Camarillo, CA, USA) were quantified by immunoassays. OPN was determined by ELISA (R&D Systems, Minneapolis, MN, USA) with intra- and inter-assay coefficients of variation being 3.2 and 5.9%, respectively^29^.

### Thiobarbituric acid reactive substances

Determination of lipid peroxidation was measured as previously described^31^. We used serum malondialdehyde (MDA) levels as an indicator of lipid peroxidation and oxidative stress. Briefly, 5 μL of serum or standard (MDA) were mixed with 120 μL of diethyl thiobarbituric acid (DETBA) 10 mmol/L and then vortexed and incubated for 1 h at 95 °C. Vials were cooled 5 min at room temperature (RT) and 360 μL of *n*-butanol were added to DETBA-MDA adducts. Samples were shaken with vortex for 1 min and centrifuged for 10 min at 1,600 *g* at RT. Then, 250 μL of supernatant were read on 96-well plates on a Fluroskan Ascent (Thermo Fisher Scientific, Waltham, MA, USA) with 535 nm and 590 nm excitation and emission wavelength, respectively.

### Statistical analysis

Data are presented as mean ± SD unless otherwise indicated. Differences in gender distribution were analyzed by χ^2^ analysis. Differences between groups were analyzed by two-tailed unpaired Student’s *t* tests. Correlations between two variables were computed by Pearson’s correlation coefficients (*r*). A *P* value lower than 0.05 was considered statistically significant. The calculations were performed using SPSS 23 (SPSS, Chicago, IL, USA) and GraphPad Prism 6 (GraphPad Software, Inc., La Jolla, CA, USA).
